# Health-related quality of life in amputees with a microprocessor-controlled prosthetic knee measured by the EQ-5D-5L

**DOI:** 10.33137/cpoj.v9i1.46403

**Published:** 2026-03-11

**Authors:** B Brüggenjürgen, A Kannenberg, C. (in memoriam) Stukenborg-Colsman, A Hahn

**Affiliations:** 1 Institute for Health Services Research and Technical Orthopedics, Orthopedic Department - Medical School Hannover (MHH) at DIAKOVERE Annastift Hospital, Hannover, Germany.; 2 Otto Bock Healthcare LP, Austin, Texas, USA.; 3 Foot Department, Orthopedic Department - Medical School Hannover (MHH) at DIAKOVERE Annastift Hospital, Hannover, Germany.; 4 Otto Bock Healthcare Products GmbH, Vienna, Austria.

**Keywords:** Amputee, Prosthetic Knee, Quality of Life, EQ-5D-5L, EQ-5D VAS, Utility, Amputation, Prosthesis, Microprocessor-Controlled Prosthetic Knee

## Abstract

**BACKGROUND:**

Enhancing health-related quality of life (HRQoL) is a fundamental objective of healthcare delivery. Individuals with amputation might encounter physical, psychological, and social challenges in daily life which might be influenced by the type of prosthetic knee provided.

**OBJECTIVE:**

To understand the experience-based HRQoL of amputees who were using a microprocessor-controlled prosthetic knee (MPK).

**METHODOLOGY:**

Amputees using an MPK for at least 3 months (C-Leg or Genium/Genium X3) participated in an online survey. In 2022 1,868 MPK user were invited to participate in the study via both desktop and mobile platforms of a German manufacturer database. HRQoL was assessed with the EuroQol 5-Dimension 5-Level (EQ-5D-5L) instrument. The data was analyzed descriptively and by measures of central tendency.

**FINDINGS:**

512 participants (19.7% female, mean age 54 years (standard deviation 12.6)) responded and fully completed the EQ-5D-5L. The leading cause of amputation was trauma (56.1%), followed by tumor (15.6%) and peripheral arterial disease (PAD, 11.3%). 18.2% reported being completely problem-free. 2.9% had a hip disarticulation, 68.0% a transfemoral amputation, 26.6% a knee disarticulation and 2.5% a bilateral amputation. The share of “no problems” was 53.3% for mobility, 88.1% for self-care, 61.5% for usual activities, 25.0% for pain or discomfort, and 67.4% for anxiety/depression. The total mean index score based on the German value set was 0.84 (maximum score 1). The total mean visual analogue scale (VAS) score was 77.4 with variations observed for age and health status.

**CONCLUSION:**

Amputees with an MPK experienced very few issues in the self-care dimension but faced more challenges in the pain/discomfort and mobility dimensions. Utilities were similar to the general German norm reporting one medical condition. MPKs enable individuals to achieve quality of life outcomes that are comparable to those observed in the general population.

## INTRODUCTION

Enhancing health-related quality of life (HRQoL) is a fundamental objective of healthcare delivery.^1^ Amputees with a prosthetic knee experience challenges that affect their quality of life (QoL).^2^ Physically, they must adapt to the prosthesis and deal with mobility limitations. Psychologically, they may face emotional distress and body image issues. Socially, they may face barriers to integration, with participation being one of the strongest determinants of QoL.^3^ Adaptation to lower-limb amputation is a multifaceted process. Health-related quality of life (HRQoL) is typically reduced initially but can be influenced by factors such as rehabilitation and perceived social support. Additionally, the presence of depression or anxiety and individual coping strategies significantly affect the adaptation trajectory, underscoring the importance of multidisciplinary interventions to optimize long-term outcomes.^4^

Assessing the QoL of patients with disabilities, especially amputees, provides valuable insights into the overall well-being and functional status of individuals with disabilities and helps healthcare professionals understand the impact of the disability.^5^ Decision-makers can use QoL-data to assess and compare the impact of interventions and - in combination with economic evaluations - guide resource allocation.^6^ The most widely utilized generic HRQoL instruments include the Short Form-36 (SF-36) and its preference-based derivative, the Short Form-6 dimensions (SF-6D), as well as the EuroQol-5 Dimension (EQ-5D).^6^ While the SF-36 offers a more detailed assessment of HRQoL, the EQ-5D is favored by health technology assessment organizations due to its simplicity and ease of use.^7^ The current EQ-5D-5L form is an expanded and more sensitive version of the EQ-5D-3L,^8^ which was also used for assessing patients with prostheses following amputation.

In Germany, about 16,500 major and 45,500 minor amputations were performed every year between 2015 and 2019.^9^ In 2019, a total of 9,015 transfemoral amputations were performed, representing procedures for which the use of a prosthetic knee joint may be indicated.^9^ Prosthetic knees should ideally provide stability during stance and agility and allow controlled flexion during swing phase. Different from non-microprocessor-controlled prosthetic knees (non-MPKs), microprocessor-controlled prosthetic knees (MPKs) control the swing phase with variable walking speeds and provide a high level of safety during the stance phase.^5^ Although manufacturers of MPKs employ different design principles, all integrate microprocessors, as seen in devices such as the C-Leg or Genium (Ottobock, Duderstadt, Germany), Rheo (Össur, Reykjavik, Iceland), and Plié (Freedom Innovations, Irvine, California, United States), with the C-Leg being the most extensively studied device.^2,10^ A recent US-based survey suggested best results for C-Leg users with regard to median mobility, quality of life, and fewer injuries.^10^ Few studies with a maximum study size of 75 participants reported impact of MPKs on QoL measured by the EQ-5D with only one study using the current EQ-5D-5L.^11–13^

Given the increasing adoption of microprocessor-controlled knee prostheses and their substantial cost implications, a comprehensive understanding of their value from the patient perspective is essential. While MPKs are widely recognized for their functional advantages, less is known about how patient demographics and usage characteristics shape HRQoL outcomes in routine care settings. Germany provides a relevant setting for such an investigation due to its structured prosthetic care system. There is currently no data on the quality of life (QoL) of amputees using an MPK measured by the EQ-5D-5L for Germany. Hence, the objective of this research was to examine the health-related QoL among individuals with amputations under real world conditions who had received a prosthetic knee controlled by a microprocessor. The present study is also designed to investigate the association between age, sex, type of prosthetic knee, and duration of MPK use and HRQoL collected with the EQ-5D-5L among lower-limb amputees using MPKs and compare it to normative German data.

## METHODOLOGY

The study employed a cross-sectional design targeting individuals with amputations who had used an MPK. The study used a German manufacturer’s (Otto Bock, Duderstadt) database, as no amputation registry is available in Germany. The inclusion criteria were a documented willingness to participate in research studies following enrollment in the manufacturer’s product registry, age 18 years and older, and a duration of MPK use (C-Leg or Genium/Genium X3) for at least 3 months. Between the fourth quarter of 2021 and the second quarter of 2022, a total of 1,868 individuals were invited to participate in the study via both desktop and mobile platforms.

Data were collected using a computer-assisted interviewing (CAI) approach, specifically through a self-administered online survey. The web-based questionnaires were administered using the Rogator survey software. The questionnaire included items addressing HRQoL and demographic characteristics, as well as domains related to prosthetic care, family, social, and occupational environment, and level of independence.

Additional domains included participation (assessed using the Reintegration to Normal Living Index), productivity (measured with the Work Limitations Questionnaire-25), self-perception (evaluated by the Amputee Body Image Scale), and participant feedback. The average completion time for the full questionnaire was 32 minutes. Participants who had incomplete responses were invited for a follow-up round with identical methodology with a two-month timeline. The analyses were based on those participants who completed the survey and responded to the QoL part.

QoL was assessed with the German EQ-5D-5L instrument (EQ-5D (euroqol.org)). The EQ-5D-5L offers several advantages over the EQ-5D-3L, including a wider range of reported health states, which also helps to reduce ceiling effects.^8^ It consists of a standardized questionnaire that measures health status across five dimensions: mobility, self-care, usual activities, pain/discomfort, and anxiety/depression. Each dimension of the 5L-version now has five severity-format response levels (“no problems” to “extreme problems”/“unable to”) that together define a total of 3,125 health state profiles. For example, a profile of 12345 would indicate no problems with mobility, slight problems with self-care, moderate problems with usual activities, severe with pain/discomfort, and extreme with anxiety/depression. In order to obtain the utility index scores ranging from 0 or lower (representing the worst health state) to 1 (representing full health), the 5-digit codes representing the health states were converted to index values, based on the German normative data.^14^ A good responsiveness of the EQ-5D-5L index values was reported recently in the diagnostic subgroup of amputees in a specialized rehabilitation population.^15^ In addition to the utility index, the EQ VAS score (ranging from 0 to 100) as a self-rated health status was analyzed separately. Both the descriptive system and the EQ visual analog scale use the day of completion (today) as a recall period.

Our study evaluated the influence of sex, age (classified by age groups), amputation cause, type of prosthetic knee and duration of MPK use on QoL. We reported **1.)** the results obtained by EQ-5D dimensions, **2.)** the German^14^ value set derived EQ-5D-5L index data and **3.)** the EQ-5D-VAS data.

Although profiles (EQ-5D health states) contain rich information on health states, they are difficult to analyze and the EuroQol Group provides no guidance on how profile data might be analyzed for statistical inference.^16,17^ Hence, we reported complete profile data on a descriptive basis and analyze the dimension data statistically with regard to influence of sex, age, type of prosthetic knee, and duration of MPK use on HRQoL. After testing for normality, statistical significance was assessed using non-parametric methods (Mann-Whitney-U-Test for sex, Kruskal-Wallis-Test for age, type of prosthetic knee, and duration of MPK use) at a p-level of 0.05. Population characteristics were reported using median, minimum, and maximum and interquartile ranges.

All data analyses were conducted with SPSS (version 29). The Benjamini-Hochberg (BH) procedure was used to control for false discovery rate in multiple hypothesis testing. Appraisals with regard to the minimally important difference for EQ-5D-5L index scores were based on McClure et al.^18^

## RESULTS

### Study Population

The invitation to participate resulted in 690 consents (response rate 37%). 564 participants closed the interview session, 126 were reminded. Finally, 531 participants completed the survey with 512 participants (27%) providing complete EQ-5D-5L data. In general, there were fewer female participants, comprising 19.7% of the respondents. The mean age of all participants was 54 years. The majority of those surveyed, totaling 56%, were either employed full-time or part-time (**Table 1**).

**Table 1:** Sample characteristics (Categorical data: n (column percent), age: mean (SD), for all continuous data: median (Minimum, maximum; Interquartile Range)).

		All	Male	Female
N		512	411	101
Age		54.0 (12.6) 56 (18, 80; 46, 63)	55.2 (12.0) 57 (20, 80; 48, 64)	48.9 (13.5) 51 (18, 74; 40, 59)
Age categories	18-34	52 (10.2)	32 (7.8)	20 (19.8)
	35-44	56 (10.9)	45 (10.9)	11 (10.9)
	45-54	123 (24.0)	91 (22.1)	32 (31.7)
	55-64	170 (33.2)	144 (35.0)	26 (25.7)
	≥ 65	111 (21.7)	99 (24.1)	12 (11.9)
Employment status	Employed	284 (55.5)	231 (56.2)	53 (52.5)
	Unemployed	207 (40.4)	161 (39.2)	46 (45.5)
	Pension	21 (4.1)	19 (4.6)	2 (2.0)
Time since amputation in years		15 (1, 68; 5, 33)	16.5 (1, 67; 6, 33)	10 (1, 68; 4, 34)
Amputation level	Hip disarticulation	15 (2.9)	11 (2.7)	4 (4.0)
	Transfemoral	348 (68.0)	276 (67.2)	72 (71.3)
	Knee disarticulation	136 (26.6)	114 (27.7)	22 (21.8)
	Transfemoral bilateral	3 (0.6)	3 (0.7)	0
	Transfemoral & Transtibial	6 (1.2)	4 (1.0)	2 (2.0)
	Knee disarticulation bilateral	2 (0.4)	2 (0.5)	0
	Knee disarticulation & Transtibial	2 (0.4)	1 (0.2)	1 (1.0)
Underlying conditions	Trauma (accident)	287 (56.1)	252 (61.3)	35 (34.7)
	Tumor (cancer)	80 (15.6)	52 (12.7)	28 (27.7)
	Peripheral artery disease	58 (11.3)	46 (11.2)	12 (11.9)
	Severe infection	23 (4.5)	14 (3.4)	9 (8.9)
	Congenital limb deficiency	15 (2.9)	9 (2.2)	6 (5.9)
	Diabetes mellitus	1 (0.2)	1 (.2)	
	Other	48 (9.4)	37 (9.0)	11 (10.9)
Duration of prosthesis use in years		15.7 (1, 67; 5.2, 33.4)	16.6 (1, 65; 5.9, 33.3)	11.0 (1, 67; 4.0, 34.4)
Prosthesis use duration categories	Below 19 years	289 (56.4)	227 (55.2)	62 (61.4)
	20-39 years	138 (27.0)	113 (27.5)	25 (24.8)
	Above 40 years	85 (16.6)	71 (17.3)	14 (13.9)
Current prosthesis	C-Leg	164 (32)	134 (32.6)	30 (29.7)
	Genium / Genium X3	348 (68)	277 (67.4)	71 (70.3)
Duration of current prosthesis use in years		4.2 (0.01, 54.9, 2.3, 7.5)	4.9 (0.1, 54.9, 2.5, 7.9)	3.2 (0.1, 23.2, 2.1, 5.9)
	≤ 1 year	31 (6.1)	23 (5.6)	8 (7.9)
	< 1-10 years	400 (78.1)	316 (76.9)	84 (83.2)
	≥ 10 years	81 (15.8)	72 (17.5)	9 (8.9)

### EQ-5D-5L Descriptive System - Dimensions

Among the five dimensions, the fewest problems were reported for self-care (11.9%) and the most for pain/discomfort (75.0%), followed by mobility (46.7%). Severe problems (at least one “5” in any dimension) were very rare (2.1%). A total of 18.2% reported being problem-free (11111). The majority of respondents (58%) gave responses not exceeding minor problems (maximum “2” in any dimension).

**Figure 1:**
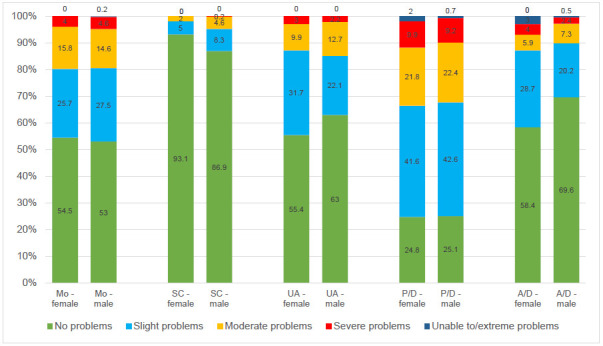
Distribution of responses (in percent) to the descriptive system of the EQ-5D-5L by sex.

Self-care (SC) was reported predominantly without any problems in both male and female amputees with a slightly lower proportion in female amputees (**Figure 1**). The highest percentage of patients with slight or moderate problems was observed in the pain and discomfort dimension (P/D). A difference between male and female was found in the anxiety and depression (A/D) dimension among those reporting problems (p = 0.03, Benjamin-Hochberg corrected (BHc) 0.17), whereas no statistically significant differences were seen in other dimensions. (p=0.84 Mo BHc 0.84; 0.81 SC BHc 0.84; 0.29 UA BHc 0.72; 0.76 P/D BHc 0.84; 0.03 A/D BHc 0.17)

**Figure 2:**
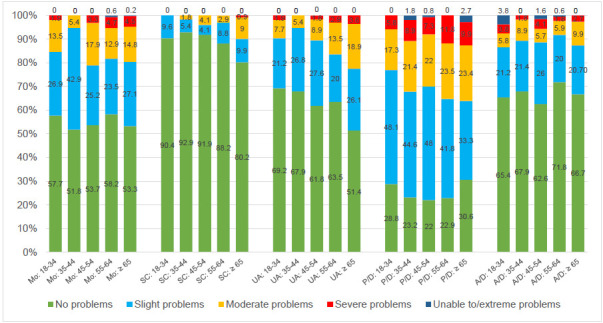
Distribution of responses (in percent) to the descriptive system of the EQ-5D-5L by age groups.

The analysis of the results by age group (**Figure 2**) revealed that amputees aged 35–44 reported the lowest impact on mobility, while the usual activities (UA) dimension showed a consistent and significant decline with increasing age. (p=0.05 Mo BHc 0.12; 0.08 SC BHc 0.13; 0.02 UA BHc 0.10; 0.34 P/D BHc 0.42; 0.79 A/D BHc 0.79).

**Figure 3:**
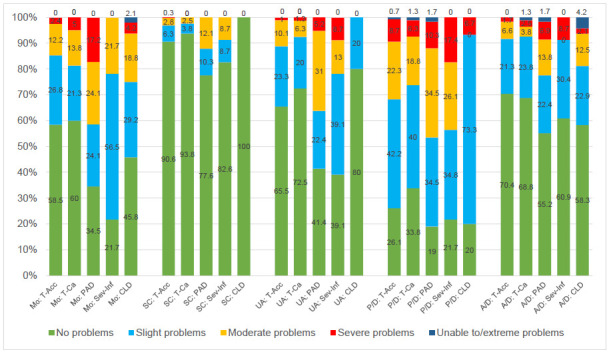
Distribution of responses (in percent) to the descriptive system of the EQ-5D-5L by cause of amputation.

The analysis of problems by cause of amputation (**Figure 3**) indicated greater impairments in both mobility and usual activities dimensions among amputees with peripheral artery disease or severe infection as the underlying etiology. Amputees with congenital limb deficiency were the only group that reported no impairments in the self-care dimension. (p=0.14 Mo BHc 0.14; 0.01 SC BHc 0.05; 0.04 UA BHc 0.06; 0.04 P/D BHc 0.06; 0.14 A/D BHc 0.14).

**Figure 4:**
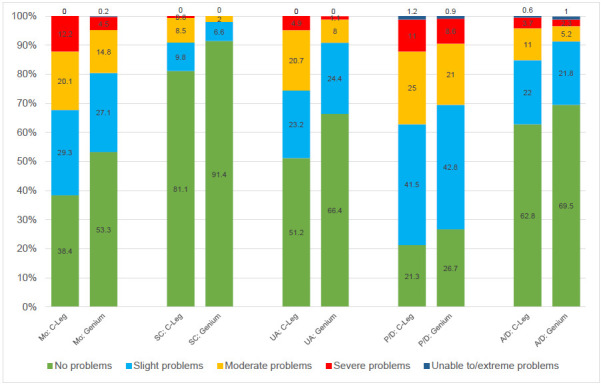
Distribution of responses (in percent) to the descriptive system of the EQ-5D-5L by type of prosthesis.

The analysis of problems by type of prosthetic knee joint (**Figure 4**) found less impairments in all dimensions for the Genium or Genium X3 than for C-Leg users with mobility, self-care and usual activities being significant prior to a potential impact of multiple testing. (p<0.01 Mo BHc 0.02; <0.01 SC BHc 0.02; <0.01 UA BHc 0.02; 0.09 P/D BHc 0.09; 0.08 A/D BHc 0.09).

**Figure 5:**
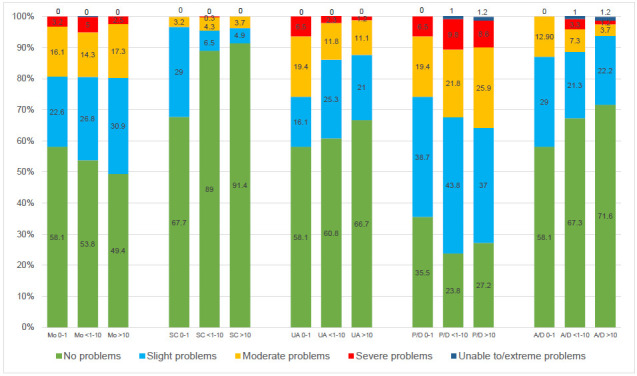
Distribution of responses (in percent) to the descriptive system of the EQ-5D-5L by duration of use of current prosthesis.

Analyses stratified by duration of use of the current prosthesis (**Figure 5**) suggested an association between shorter prosthesis use duration and higher reported impairments in the anxiety/depression dimension. (p=0.84 Mo BHc 0.84; 0.81 SC BHc 0.84; 0.29 UA BHc 0.72; 0.76 P/D BHc 0.84; 0.03 A/D BHc 0.15).

### EQ-5D-5L Value Set-Based Index

The average generated index values, categorized by both sex and age, ranged from 0.82 to 0.86 with a study population mean of 0.84 (**Table 2**). These differences are considered not clinically meaningful, except for the comparison between the age groups “18-34” and “>65”, however, the difference was not statistically significant (p= 0.14).

**Table 2:** Average Index value - total and by sex and age (IQR = interquartile range).

		N	Median	Mean	Minimum	Maximum	IQR 25	IQR 75	p
Total (participants provided complete EQ-5D-5L data)		512	0.91	0.84	−0.08	1	0.81	0.94	
Sex	Male	411	0.91	0.85	−0.08	1	0.81	0.94	0.48 (U-Test)
	Female	101	0.89	0.82	−0.04	1	0.82	0.94	
Age categories	18-34	52	0.94	0.86	0.16	1	0.83	0.97	0.55 (Kruskal-Wallis-Test: H)
	35-44	56	0.92	0.86	0.28	1	0.82	0.94	
	45-54	123	0.90	0.85	−0.04	1	0.83	0.94	
	55-64	170	0.89	0.84	0.18	1	0.82	0.94	
	> 65	111	0.89	0.82	−0.08	1	0.73	0.97	

### EQ-5D-5L VAS

**Figure 6:**
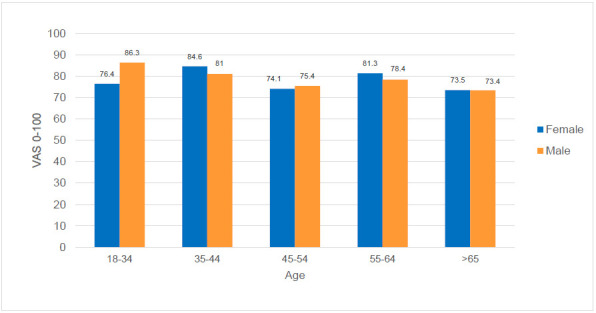
Mean VAS scores by age group and sex.

A total of 511 respondents provided VAS “health today” data. Fourteen patients indicating a “0” were excluded due to implausibility as the QoL change question indicated an improvement and/or index values were greater than 0.2 (n=497). The mean VAS score was similar for male and female amputees (77.4 male, 77.4 female; p=0.82), with female being 6.3 years younger (**Figure 6**).

The VAS means varied depending on whether there were problems reported in the individual dimensions (p<0.001): the VAS mean for participants in the problem-free health state (11111) was 89.6 representing 18.2%. For participants who reported at least one problem, the mean VAS score was 14.9 points lower (74.7). The share of participants with at least one problem (81.8%) was greater than the share of people who reported being problem-free (18.2%). With regard to age, the mean VAS score of the oldest group of participants was 9.2 points lower than the mean VAS score of the youngest group (p<0.01).

## DISCUSSION

Our comprehensive analysis of the health status of a large population of German amputees did yield several important findings. The majority of participants in our survey indicated either no limitations (18.2%) or only minor limitations (58.0%) across the five dimensions of the EQ-5D-5L. Only a small percentage (2.1%) reported experiencing extreme problems in at least one dimension. The proportion of female participants was 20%, which is similar to the 25% female representation among the 178 participants in a recent C-Leg study and the 17% female representation in the reported Genium routine trial fitting data.^10,19^ The reported share of all amputations in female patients in Germany stands at 32%.^9^ We report a mixed real world population with a vascular etiology of 11.3%, which is in the range of other observational studies such as the Genium trial fitting database with 6%, or a MPK comparison at 29.8%.^10,19^ Using the German EQ-5D-5L value set, female amputees reported a slightly lower health-related quality of life compared to men, though this difference was neither statistically significant nor clinically meaningful.^18,20^

### Main Findings

#### EQ-5D-5L Descriptive System - Dimensions

Two extensive EQ-5D-5L normative datasets for Germany were published.^21,22^ The phone survey study (4998 German adults, 2014) by Grochtdreis et al.^21^ reported a higher percentage (30.6%) of respondents with no problems, while the proportion of individuals with reporting no or only minor limitations was somewhat lower (35.8%) than that in our survey (58.0%). Next to the condition-related differences, other aspects such as age may play a role. Our survey participants had a mean age of 54 years compared to 50.7 years in the weighted normative data. Age as an explanatory factor is further supported by the omnibus survey study (2040 evaluable participants, 2012 and 2015) conducted by Huber et al., which had a mean participant age of only 47 years. They reported an even significantly higher percentage (64.3%) of respondents indicating no problems on the EQ-5D-5L scale.

Notably, the low occurrence of self-care issues in our study was comparable to the findings reported by Huber et al., with percentages of 11.9% and 7%, respectively.^22^ Although the dimensions with the highest number of problems (pain/discomfort) and the second-highest number of problems (mobility) were similar to Huber et al.'s findings, the actual prevalence of problems differed considerably from that in the amputees who participated in our study. Pain/discomfort was reported as being problem-free in only 25.0% in our study, while it was reported in 71.1% in Huber et al.'s study. Mobility without problems was reached by 46.7% vs. 81.3%. Interestingly, reported problems in the Usual Activities dimension were age-related, with severity of problems increasing with age, while peripheral artery disease and severe infection as causes of amputation also impacted the Mobility dimension. Mobility, Usual Activities, and Self-Care differed based on the type of prosthesis, with Genium or Genium X3 demonstrating superior outcomes.

#### EQ-5D-5L Value Set-Based Index

Mean index values in our survey population were marginally lower than those reported for the German normative population for individuals with at least one medical condition, with no statistically significant difference observed. (male: 0.85 vs. 0.91 and female: 0.82 vs. 0.89).^21^ The comparatively high values may be attributed to the exclusive survey participation of amputees using MPKs, suggesting that they were already experiencing the benefits of advanced MPK technology.^10^ Another possible explanation is that patients perceived specific dimensions of health conditions significantly different than the general public because they view mobility and self-care difficulties as comparably of lesser burden, while experiencing more challenges with pain/discomfort and anxiety/depression.^23^ This specific pattern pertains specifically to the pain and discomfort dimension that resembles what we observed in our study. However, it should be noted that pain experienced by individuals with lower-limb amputation may be influenced by prosthetic-related factors - such as socket design, suspension system, and residual limb condition - rather than originating only from the knee joint.

#### EQ-5D-5L VAS

In addition to the dimensions and the index of the EQ-5D-5L, we analyzed the VAS results of the instrument. Although the VAS emphasizes the patient’s perspective, the use of the VAS score as a utility measure is often criticized - mainly due to methodological considerations.^24^ However, the VAS is also reported to be more advantageous with regard to simplicity, reliability, validity, and practicality in health state valuation; hence, these data might be supportive when feasibility and acceptability are of major concern.^24^

Our VAS total mean of 77.4 was lower than that in the omnibus survey study by Huber et al. which reported a VAS total mean of 85.1;^22^ however, our VAS total mean was greater than the reported 71.6 by Grochtdreis et al. in a representative sample.^21^ International studies have reported comparable normative VAS values of the EQ-5D-5L for the general population: a Spanish study with a high percentage of females (54%) reported a mean VAS of 75.7, an English study a mean VAS score of 78.4, and a Canadian study a mean VAS score of 79.0.^8,25,26^

Huber et al. reported German VAS mean data stratified by age and sex, starting with the highest values in those aged 14-19 (98.2. male vs. 94.7 female) and constantly decreasing to 71.7 and 70.8, respectively, in the 70-79 age group.^22^ Interestingly, similar values of 73.4 vs. 73.4 were reported for our group aged 65 years and older. However, different from Huber et al. female amputees in the 18– to 34-year-old age group had a considerably lower VAS mean of 76.4 points, which might indicate a stronger impact in the younger age subgroup perhaps due to more or not-yet adapted participation and integration issues following amputation. Furthermore, different from a constant decline increasing with age, we observed peaks in the 35- to 44 and 55- to 64 age groups.

#### MPKs vs. non-MPKs

HRQoL utilities in individuals with lower limb amputation differs with the technical features of knee prostheses, with microprocessor-controlled knees providing better QoL.^27^ However, few data is available with regard to utilities for those not having been fitted with an MPK. Guirao reported a utility of 0.79 for patients in Spain fitted with a mechanical prosthesis (non-MPK) based on mapped SF-36 data, whereas Cutti et al. reported a utility of 0.68 for patients with non-MPK in Italy.^28,29^ For MPKs, Cutti et al. reported data from 70 patients using the C-Leg, with most problems being reported in the pain or discomfort dimension (71.4% reporting problems in the EQ-5D-3L), which was also the most relevant dimension in our study (75% with problems).^28^

### Policy Implications and Reimbursement Application

Our findings indicate a high HRQoL among amputees using an MPK, suggesting a generally high standard of healthcare provision for MPK users in Germany. However, due to the absence of a fully established German amputation registry, it is not-yet possible to determine the prevalence of MPK fittings in Germany and whether more patients could benefit from an MPK prosthesis.

The long duration of MPK prosthesis use in our study must be considered when applying our results in economic evaluations, as the beneficial effects of a fully completed adaptation period may have positively influenced the results. To enhance the applicability of our findings in health economic assessments for reimbursement, future research should incorporate similar surveys including transfemoral amputees prior to MPK fitting and non-MPK users. This would facilitate a more comprehensive understanding of the impact of MPK use on quality of life as measured by the EQ-5D.

### Strengths

Our analysis offers a detailed insight into how amputees with an MPK perceive their disability in relation to health-related quality of life (HRQoL), contributing to a better understanding of their self-perception. Compared to previous studies with smaller sample sizes, the large number of participants in our study enabled robust stratified analyses. Furthermore, our findings are based on individual real-world data from affected individuals in Germany, enhancing the relevance and applicability of the results.

### Limitations

A potential limitation may have arisen from the selection of a manufacturer database that lacked MPKs of other manufacturers. Although microprocessor-controlled knee prostheses are frequently considered a homogeneous device category, functional differences exist between individual MPK systems, with the C-Leg offering certain advantages in a previous published comparison.^10^ As Ottobock is a leading provider of MPKs for German amputees, the dataset is likely representative of the broader population of MPK users in Germany. A potential bias could have arisen from a response rate of 37%, which is near the 45% observed in recent patient online surveys.^30^ However, given that the age distribution, female representation, and reasons for amputations align with those found in other amputee studies, any resulting bias is likely minimal.^10,19^ Owing to the absence of comprehensive registries encompassing the full population of amputees, the survey was unable to include a control group. Additionally, all amputees in the database were digitally proficient, as they used a cockpit app for communication with the manufacturer. A further limitation of this study is that a few patients with implausible VAS scores had to be excluded.

## CONCLUSION

Patients who used microprocessor-controlled prosthetic knees after amputation reported a high level of health-related quality of life (HRQoL) based on EQ-5D-5L assessments suggesting a beneficial impact of the MPK technology. They experienced very few issues in the self-care dimension but more challenges in the pain/discomfort and mobility dimensions. The HRQoL and utilities of amputees using an MPK were found to be similar as those of the general German population reporting one dimension-related problem. Our VAS mean data from the EQ-5D-5L showed notably greater scores compared to published non-microprocessor knee data, with the share of problem-free individuals being comparable to normative data. Microprocessor-controlled knees enable individuals to achieve quality of life outcomes that are comparable to those observed in the general population. Further research is required to enable comparisons with non-microprocessor-controlled knees and to establish standardized frameworks for evaluating prosthetic outcomes, including the development of comprehensive databases and longitudinal follow-up studies.

## Data Availability

The data that support the findings of this study are available from Ottobock but restrictions apply to the availability of these data, which were used under license for the current study, and so are not publicly available. Data are available from the authors upon reasonable request and with the permission of Ottobock.
